# Comparison of an In-Person versus a Virtual Interprofessional Education Activity Focused on Professional Communication

**DOI:** 10.3390/pharmacy9020111

**Published:** 2021-06-15

**Authors:** Tracey DelNero, Deepti Vyas

**Affiliations:** 1 School of Health Sciences, University of the Pacific, 3200 Fifth Ave., Sacramento, CA 95817, USA; tdelnero@pacific.edu; 2 School of Pharmacy, University of the Pacific, 3601 Pacific Ave., Stockton, CA 95211, USA

**Keywords:** SBAR, interprofessional education, pharmacy student, physician assistant student, professional communication, TeamSTEPPS, virtual

## Abstract

Early provision of interprofessional education (IPE) is imperative to ensure effective communication between healthcare professionals. However, there are several barriers to offering adequate IPE, including space restrictions and lack of human resources, prompting exploration of alternative modalities. In 2019, an IPE activity was offered in person with 213 pharmacy and 45 physician assistant (PA) students participating in one-on-one team huddles focusing on managing an acutely ill patient. In 2020, the same IPE activity, including 194 pharmacy and 45 PA students, was offered virtually. Peer evaluations, an attitudes survey, and confidence surveys were administered to evaluate the impact of the IPE activity. A student *t*-test and descriptive statistics were utilized to analyze the data. On average, PA students in the virtual group rated their peers higher than PA students in the in-person group, with little difference in the pharmacy peer evaluation of their PA partner. Ninety percent of pharmacy students and 91% of PA students in the virtual group felt that “they learned something new regarding therapeutic management” from their partner versus 84% of pharmacy and 81% of PA students in the in-person group. In conclusion, using a virtual modality for a communications-focused IPE was not detrimental to student attitudes and did not adversely affect peer perceptions.

## 1. Introduction

Interprofessional communication is a critical step in improving patient outcomes and moving healthcare systems from the traditional silo approach to a more integrated team-based approach to patient care [1]. Healthcare professionals (HCPs) must have confidence in their communication abilities to serve as effective health care team members. It is also essential for HCPs to build trust and seek and engage their team members in shared problem-solving [2]. Early preparation of students is vital in developing these abilities, allowing graduates to step into the healthcare system with an adequate understanding of patient care provision within a team-based context. Preparing HCP students within an interprofessional education (IPE) context can allow them to learn from and with each other early on in their careers and develop good interprofessional communication skills [1]. Poor communication can result in substandard care and patient harm [2]. The reality of today’s healthcare environment is that providers are responsible for several patients at a time and can easily confuse one patient with another. Other barriers to communication include high-stress working environments, rapidly changing medical teams, and lack of structure in professional communication [2,3,4,5,6,7]. One systematic approach to optimize teamwork is the Team Strategies and Tools to Enhance Performance and Patient Safety (TeamSTEPPS^®^), which consists of various communication tools, such as the SBAR (situation, background, assessment, recommendation) oral communication technique, team huddle, call-outs, and check-back [8]. Using TeamSTEPPS^®^ tools can allow HCPs to orient other HCPs to the patient and provide the most relevant information to justify treatment recommendations. The SBAR offers a systematic framework for team huddles to ensure that the most relevant information is provided to justify recommendations regarding patient management [9]. Several studies have provided insight into student training on the SBAR tool [3,4,5,6,7]. Kostoff and colleagues described an IPE activity that focused on using the SBAR in phone sessions involving nursing and pharmacy students. Attitudes on the Interprofessional Collaborative Competencies Attainment Survey (ICCAS) improved after this activity and written reflections indicated positive improvements in their communication abilities [3]. In a uniprofessional study, Brusti-Sisti and colleagues found that SBAR training improved pharmacy student confidence and ability to relay information systematically [4]. Similarly, Shrader and colleagues found that pharmacy student communication skills significantly improved after SBAR-focused simulations [5]. However, several studies have elucidated that training students using the TeamSTEPPS^®^ tools can be resource- and time-intensive [9,10,11,12,13]. Utilizing alternative strategies, such as virtual or remote IPE activities, can mitigate some of the barriers illustrated in the literature. 

In a study explicitly geared toward the mode of delivery, Sherman and colleagues found no differences in student attitudes when comparing a telephone-based versus video-conferencing-based IPE activity, concluding that the mode of delivery does not necessarily impact student outcomes [14]. A similar study by Djukic found that a virtual IPE mainly had positive outcomes except for medical student attitudes revolving around team value [15]. On the other hand, Lempicki and colleagues who described the impact of a web-based versus face-to-face IPE focused on professional communication with standardized patients (SPs) found slightly lower scores on both the Indiana University Simulation Integration Rubric and the communication assessment tool-team for the web-based group. Unfortunately, the relatively small subject size made it challenging to make any meaningful deductions from this study [16]. In general, there is a dearth of studies directly comparing in-person versus virtual IPE to determine if virtual modalities are just as successful in improving outcomes as traditional face-to-face activities. The purpose of this study was to compare students’ attitudes and peer perceptions regarding an IPE activity, focusing on TeamSTEPPS^®^ communication tools, conducted in person versus that conducted virtually. 

## 2. Materials and Methods

The University of the Pacific offers a longitudinal IPE curriculum involving pharmacy and physician assistant (PA) students delivered as a series of experiences offered throughout the didactic curriculum. Both programs are accelerated and are all year-round. The pharmacy didactic curriculum occurs over six semesters beginning in the fall, while the PA didactic curriculum is 3.5 semesters long, beginning in the spring of each year. The fifth activity in the IPE series occurs in the fall semester and involves second-year pharmacy and 1st-year PA students. Interprofessional teams consisting of one PA and one pharmacy student collaborate on the care of an acutely hospitalized patient using TeamSTEPPS^®^ communication concepts. The specific learning objectives of the IPE activity are to use TeamSTEPPS^®^ concepts, including the SBAR tool, team huddle, call-outs, and check-backs to collaborate on the care of an acutely ill patient.

This study compares outcomes from two academic years. The data included were derived from the IPE activity in the fall of 2019 and 2020. In 2019, the IPE activity was offered in person with 194 pharmacy students and 45 PA students participating in one-on-one simulations. A year later, the same training and IPE activity, including 194 pharmacy and 45 PA students, occurred virtually via videoconferencing technology. The subject matter and patient case were the same each year. The sole difference was the mode of delivery for educating students on TeamSTEPPS^®^ concepts and the IPE activity. Due to the COVID-19 pandemic, starting in the spring semester of 2020, the pharmacy curriculum was offered entirely online while the PA students continued to meet in person for select coursework. The 2020 pivot required faculty members to identify alternate strategies for offering IPE.

### 2.1. Training on TeamSTEPPS Concepts

Preliminary training was delivered using a YouTube video that illustrated strategies to communicate with another HCP using TeamSTEPPS^®^ tools. One week before the IPE, to prepare pharmacy students for the IPE activity, third-year pharmacy students on clinical clerkships were recruited to simulate a provider. For the training session, course faculty members assigned an inpatient electronic health record (EHR) of a patient presenting with a chief complaint of an acute gastrointestinal bleed due to a peptic ulcer. Within the computerized provider order entry (CPOE) system, the provider had ordered medications for the acute management of the patient. Some of these orders were not optimal for the patient. Each pharmacy student was required to complete an independent review of the patient scenario and develop a modified treatment plan. Pharmacy students then participated in a one-on-one simulation with the near-peer using TeamSTEPPS^®^ concepts. Each simulated encounter with the near-peer lasted about 20 min. The 3rd year (near-peer) students received a grading rubric, which assessed the students’ use of the SBAR tool and other communication techniques. A freeform comment section was also built into the rubric to provide targeted comments about the student’s performance. Traditionally, this near-peer training occurs in person with 3rd-year students coming to the campus for the day. In 2020, this near-peer training occurred via videoconferencing technology. During their first semester of enrollment, the PA students learned the TeamSTEPPS^®^ principles and utilized the SBAR tool frequently in other educational activities. Before the IPE event, the PA students reviewed the same YouTube video but did not participate in the near-peer training activity as the PA students used the SBAR tool throughout their didactic training in other peer educational activities. 

### 2.2. IPE Activity

For the IPE activity, course faculty members assigned an inpatient EHR case on cirrhosis and associated complications. All students received education on cirrhosis and its complications previously. Within the computerized provider order entry (CPOE) system, the previous provider had ordered medications for the acute management of the patient. Some of these orders were either without any indication, not the optimal choice given the patient’s presentation or had dosing errors. Opportunities to optimize the medication regimen existed. Students from both professions were to identify the errors and recommend corrective action. Each pharmacy and PA student individually reviewed the case and developed a modified therapeutic plan. This part of the exercise was 90 min. 

**Figure 1:**
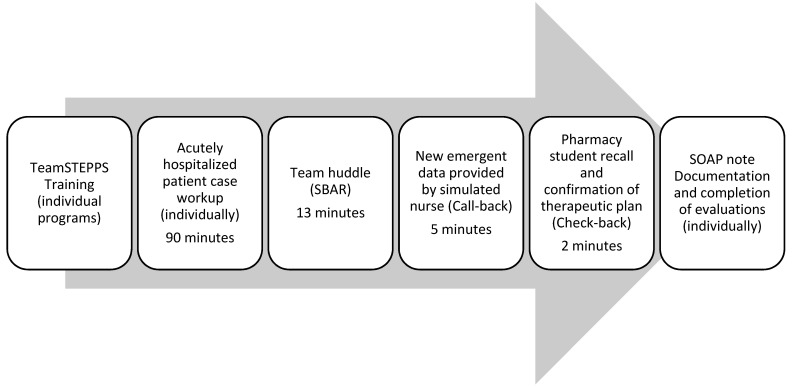
Interprofessional Education Activity Format with Pharmacy and Physician Assistant Students. TeamSTEPPS^®^ = Team Strategies and Tools to Enhance Performance and Patient Safety; SBAR = situation, background, assessment, and recommendation; SOAP = subjective, objective, assessment, and plan.

In 2019, students completed one-on-one team huddles in a physical classroom. Due to space limitations, several teams were in the same classroom. IPE facilitators instructed the pharmacy students to use the SBAR tool for the team huddle. During the huddle, both the PA and pharmacy student received one additional piece of data from a standardized colleague (nurse) to simulate a call-out. The faculty instructed both the PA and pharmacy students to develop a plan based on the new emergent data. Both the team huddle and call-out allowed students to learn with and from each other. Due to the discrepancy in the pharmacy to PA student ratio, each PA student participated in four to five one-on-one encounters while each pharmacy student participated in only one encounter. PA students remained in each classroom while pharmacy students rotated in and out. Each encounter’s call-out contained a different piece of new data to facilitate continued PA student engagement in the activity. Pharmacy students then used the check-back to clarify the treatment plan. After their encounter, students documented their care of the patient by writing a SOAP note, including any new therapy that arose during the team huddle (Figure 1). Students also completed all evaluations at the end of the IPE activity. In 2020, the IPE activity remained the same except that videoconferencing breakout rooms were utilized for the TeamSTEPPS. Typed “broadcast” messages relayed the call-outs. The same case was used in 2020 and has been utilized for this IPE activity since 2016.

### 2.3. Evaluation

In both 2019 and 2020, students completed a peer evaluation of their teammate using a four-point Likert scale ranging from 4 = excellent and 1 = unsatisfactory. PA and pharmacy students also completed an attitudes survey to determine their perception of the IPE activity on a scale of ‘Yes or No”. Pharmacy students also completed a three-item pre and post-confidence survey using a five-point Likert scale ranging from 5 = very confident and 1 = not at all confident. PA students completed a two-item survey measuring the impact of the IPE activity on their “confidence in communicating concerns regarding therapeutic plans” and the “benefit of the activity on increasing their medical knowledge” on a five-point Likert scale ranging from 5 = definitely enhanced and 1 = definitely worsened. T-tests were used to assess differences between the 2019 and 2020 data, the scores from the rubric on individual items and total SBAR scores, and student confidence levels for each item. Student *t*-tests were used due to the large sample size and the normal distribution of the data. A *p*-value of <0.05 was used to determine statistical significance for all statistical methods. Descriptive statistics were used to describe the attitudes survey. Excel^®^ was used for data storage and statistical calculations. In addition, a quantitative analysis of space and faculty resources was conducted to determine any differences in resource needs.

The institutional review board (IRB) at the University approved this study.

## 3. Results

Ninety percent of pharmacy students (*n* = 174, RR = 100%) and 91% (*n* = 41, RR = 100%) of PA students in the 2020 group (virtual) felt that “they learned something new regarding therapeutic management” from their partner versus 84% (*n* = 179, RR = 100%) of pharmacy and 81% (*n* = 36, RR = 100%) of PA students in the 2019 group (in-person). Ninety-six percent (*n* = 229, RR = 100%) of the 2020 group felt this IPE was beneficial to their learning versus 94% (*n* = 242, RR = 100%) in the 2019 group. On the peer evaluation by pharmacy students (of their PA partner), there was no statistically significant difference on any ratings between the virtual versus in-person evaluations except for the item “rate this student’s ability to convince you to change the patient’s medication plan” (3.61/4 in 2019 versus 3.76/4 in 2020, *p* < 0.01, Table 1).

On the peer evaluation by PA students, there was a significant difference on seven items, with the virtual group providing higher peer ratings than the in-person group. (Table 2).

On the pre/post confidence survey, the 2019 pharmacy group had higher confidence levels than the 2020 group on all three items. On the item, “what is your confidence level in using the SBAR method to communicate with a provider” the average on the 2019 in-person group was 3.94/5 versus 3.79/5, *p* < 0.01. On the student’s confidence in relaying medication recommendations to a provider, the 2019 average was 4/5, and the 2020 average was 3.83/5, *p* < 0.01. On the final question of confidence in defending medication changes to a provider, the 2019 average was 3.74/5 versus 3.65/5 in 2020, *p* < 0.01. On the PA student surveys (response rate 91% for 2019 and 100% for 2020), there was no difference between the in-person and the virtual group, with both groups reporting that the IPE activity “definitely enhanced or enhanced” their confidence and medical knowledge.

On the resource needs assessment, the in-person IPE activity required three large classrooms and eight-room monitors (including three standardized colleagues for the call-outs). The virtual activity required two facilitators to troubleshoot technology issues and to answer student questions.

## 4. Discussion

This study compared student peer evaluations and attitudes after an IPE event offered either virtually or in person. Data analysis evaluated if the mode of delivery impacted student ratings of their peers and their attitudes regarding the utility of an IPE event. The virtual versus in-person data analysis revealed surprising results, with PA students providing higher peer ratings for the virtual IPE activity versus the in-person activity. It is unclear why this was the case as the 2020 group had to pivot to online learning due to the COVID-19 pandemic and negative attitudes were expected compared to the pre-pandemic attitudes. In addition, overall attitudes regarding the impact of the IPE activity on their learning were also more positive in the virtual group versus the in-person group. A higher percentage of students in the virtual group felt something new from their partner versus the in-person group. It is possible that the use of videoconferencing provided a more comfortable environment than the physical space, which may have been fraught with noise issues and congestion as there were several teams per classroom. Another possibility is that the 2020 PA group had overall more positive attitudes than their 2019 peers. This may have also led to higher pharmacy peer evaluation on the item “rate the student’s ability to convince you to change the patient’s medication plan”. Interestingly, on the 2020 peer evaluation by PA students of the pharmacy students, the seven items that were statistically better were related to communication, including the student’s ability to describe the situation and background, using appropriate medical language, accurately resolving any questions and respect of the partner. It is possible that the pharmacy students were more comfortable in the virtual environment than the in-person environment and this impacted their communication abilities. Other possibilities are that the distractions in the physical space may have affected the 2019 pharmacy student confidence versus those in the quiet virtual space. Despite higher peer evaluations, pharmacy students reported lower confidence in the 2020 group versus the in-person group. The discrepancy between high peer evaluations and low pharmacy student confidence is curious. It is unclear why the group has lower levels of self-efficacy. Future research would benefit from a better study design, such as a randomized trial within the same class year instead of comparing two different class years. However, it was encouraging that both the 2019 and 2020 students had high peer ratings (>3/4), suggesting that most students utilized the TeamSTEPPS^®^ concepts effectively. Our study was in contrast to some of the other studies, which found overall lower ratings for virtual IPE events versus in-person events [16]. Our study offers some support for using virtual modalities for IPE as there was little difference between the two modalities in terms of peer assessments and student attitudes. As to be expected, the virtual IPE activity required fewer human and space resources, which is encouraging for programs that do not have adequate resources for IPE delivery. However, this study had some limitations. The confidence surveys administered to the PA students were different from those in the pharmacy program. Therefore, we have no comparative data on student confidence levels. The surveys differed as each profession had different tasks during the IPE activity. Pharmacy students were required to use the SBAR tool to make their recommendations, while the PA students were the receivers of the information. Another limitation of this study is that the pharmacy school had a new curriculum in place for the 2020 class and some coursework differed from the 2019 class, which could have impacted student skills and affected the results of this study. In addition, this study did not utilize any validated interprofessional tools, such as the ICCAS or SPICE-R survey, preventing the authors from making any conclusions regarding the utility of this IPE event on interprofessional attitudes. Future studies shall include the use of the ICCAS to provide more specific information on interprofessional attitudes. In addition, due to the sudden pivot, we were unable to measure student attitudes regarding their preference for in-person versus virtual IPE events, which would have provided valuable data for instructors looking to offer virtual IPE activities. Regardless of these limitations, this study offers valuable evidence that virtual modalities are not detrimental to student attitudes and peer perceptions. A one-on-one model utilizing videoconferencing tools is a model that other programs could adapt for delivering IPE.

## Figures and Tables

**Table 1:** Peer Evaluation by Pharmacy Students.

Rubric Item	2019 Score Mean (SD) (*n* = 213)	2020 Score Mean (SD) (*n* = 194)
Accuracy of the clinical information provided by the PA student	3.67 (0.49)	3.72 (0.44)
Respected your authority and encouraged shared decision making	3.78 (0.49)	3.81 (0.4)
Used appropriate medical terminology	3.79 (0.4)	3.81 (0.4)
Engaged in consultation	3.76 (0.47)	3.79 (0.4)
Appeared confident when questioned on his/her recommendation	3.71 (0.49)	3.77 (0.43)
Accurately resolved any questions that you had	3.70 (0.5)	3.79 (0.4)
Ability to convince you to change the patient’s medication plan	3.61 (0.55)	3.76 (0.47) *

Questions stem: Rate the student on a scale of 1 to 4 with being 4 = Exceptional; 3 = Satisfactory; 2 = Somewhat Satisfactory; 1 = Unsatisfactory; * Indicates a *p*-value < 0.01.

**Table 2:** Peer Evaluation by Physician Assistant (PA) Students.

Rubric Item	2019 Score Mean (SD) (*n* = 45)	2020 Score Mean (SD) (*n* = 45)
The pharmacy student described the situation (S) to you (the provider)	3.44 (0.77)	3.62 (0.66) *
The pharmacy student provided background (B) to familiarize you (provider) with the patient	3.46 (0.75)	3.61 (0.63) *
The pharmacy student provided his/her own assessment (A) of the patient	3.54 (0.7)	3.61 (0.61)
The pharmacy student provided a recommendation (R) for managing the patient	3.59 (0.6)	3.66 (0.52)
Accuracy of the clinical recommendation	3.48 (0.67)	3.52 (0.61)
The pharmacy student respected your authority and encouraged shared decision making	3.65 (0.6)	3.82 (0.4) *
Used appropriate medical terminology	3.62 (0.6)	3.78 (0.4) *
The pharmacy student was engaged in the consultation	3.64 (0.6)	3.79 (0.4)
Maintained eye contact	3.58 (0.64)	3.65 (0.6)
Appeared confident when questioned on his/her recommendation	3.40 (0.8)	3.49 (0.7)
Accurately resolved your questions	3.46 (0.7)	3.68 (0.5) *
Was the pharmacy student able to convince you to change the patient’s medication regimen?	3.50 (0.7)	3.69 (0.5) *
How did the pharmacy student grade him/herself?	3.14 (0.6)	3.22 (0.6) *

Questions stem: Rate the student on a scale of 1 to 4 with being 4 = Exceptional; 3 = Satisfactory; 2 = Somewhat Satisfactory; 1 = Unsatisfactory; * Indicates a *p*-value < 0.01.

## Data Availability

Raw data is available here: https://docs.google.com/spreadsheets/d/1DOiNy3qQ-rvlHsm7wLAtwyqJC3FdaGgjkfqepgbW9RE/edit?usp=sharing.
